# Global change impacts on cacti (Cactaceae): current threats, challenges and conservation solutions

**DOI:** 10.1093/aob/mcad040

**Published:** 2023-03-21

**Authors:** Kevin R Hultine, Tania Hernández-Hernández, David G Williams, Shannon E Albeke, Newton Tran, Raul Puente, Eugenio Larios

**Affiliations:** Department of Research, Conservation and Collections, Desert Botanical Garden, Phoenix, AZ 85008, USA; Department of Research, Conservation and Collections, Desert Botanical Garden, Phoenix, AZ 85008, USA; Department of Botany, University of Wyoming, Laramie, WY 82071, USA; Wyoming Geographic Information Science Center, University of Wyoming, Laramie, WY 82071, USA; Center of Tree Science, Morton Arboretum, Lisle, IL 60532, USA; Department of Research, Conservation and Collections, Desert Botanical Garden, Phoenix, AZ 85008, USA; Programa Educativo de Licenciado en Ecología, Universidad Estatal de Sonora, Hermosillo, Sonora 83100, México

**Keywords:** CAM photosynthesis, *Carnegiea gigantea*, invasive grasses, nurse plants, rooting depths, Sonoran Desert, *Stenocereus thurberi*, stem water storage

## Abstract

**Background:**

The plant family Cactaceae provides some of the most striking examples of adaptive evolution, expressing undeniably the most spectacular New World radiation of succulent plants distributed across arid and semi-arid regions of the Americas. Cacti are widely regarded for their cultural, economic and ecological value, yet they are also recognized as one of the most threatened and endangered taxonomic groups on the planet.

**Scope:**

This paper reviews current threats to species of cacti that have distributions in arid to semi-arid subtropical regions. Our review focuses primarily on four global change forces: (1) increases in atmospheric CO_2_ concentrations; (2) increases in mean annual temperatures and heat waves; (3) increases in the duration, frequency and intensity of droughts; and (4) and increases in competition and wildfire frequency from invasion by non-native species. We provide a broad range of potential priorities and solutions for stemming the extinction risk of cacti species and populations.

**Conclusions:**

Mitigating ongoing and emerging threats to cacti will require not only strong policy initiatives and international cooperation, but also new and creative approaches to conservation. These approaches include determining species at risk from climate extremes, enhancing habitat quality after disturbance, approaches and opportunities for *ex situ* conservation and restoration, and the potential use of forensic tools for identifying plants that have been removed illegally from the wild and sold on open markets.

## INTRODUCTION

Approximately 30 plant lineages are distinguished as succulent, but of these only a small subset are species rich and ecologically important in arid and semi-arid ecosystems throughout the globe (Arakaki *et al.*, 2011). Among the most notable of these groups are cacti (Cactaceae), which include the most spectacular New World radiation of succulent plants, with ~1850 species distributed throughout arid and semi-arid regions within an expansive native range from throughout southern Canada to the Patogonian steppe in Argentina (Gibson and Horak, 1978; Mourelle and Ezcurra, 1996; Pinkava, 1999; Anderson, 2001; Cota-Sánchez, 2002; Arakaki *et al.*, 2011). Cacti are widely regarded for their cultural, economic and ecological value and often serve as important foundation species supporting numerous and highly specific pollinators, seed dispersers and frugivores (Alcorn *et al.*, 1961; Fleming *et al.*, 1996; Mellink and Riojas-López, 2002; Wolf and Martínez del Rio, 2003). To persist in harsh, arid environments, cacti have evolved many unusual traits, including a thick, waxy cuticle that restricts excessive water loss, a reduction in leaf size and duration, the modification of leaves into spines combined into short shoot systems termed areoles (Mauseth, 2006) and the exhibition of crassulacean acid metabolism (CAM), a carbon-concentrating photosynthetic pathway allowing plants to acquire CO_2_ at night when water loss from photosynthetic tissues is minimized (Nobel, 2003). Among the most recognizable features of cacti are their photosynthetic stems, which, in many species, store massive amounts of water and other resources to support growth, reproduction and survival during hot and dry conditions. The remarkable diversity in stem geometry and size, and in growth form in general (e.g. Hernández-Hernández *et al.*, 2011), displayed within and among species makes cacti unique among plants in terms of their physiology and morphology.

Superimposed on the already stressful conditions in which many cacti thrive are a wide range of threats caused largely by, among other factors, the illegal collection of live plants and seeds for horticultural trade, intensive land clearing and development, introduction and invasion of exotic species, increased exposure to wildfires and climate change (Goettsch *et al.*, 2015; Pillet *et al.*, 2022). The impact of these threats has the potential to be dramatic and devastating for some cacti species. Of the 1477 species of cacti evaluated by the International Union for the Conservation of Nature (IUCN), 31 % are listed as either near threatened, vulnerable, endangered or critically endangered, with ≥76 % of these species currently in decline (IUCN, 2014; Goettsch *et al.*, 2015; Hultine *et al.*, 2016*a*). A more recent assessment indicates that ≤90 % of all species of cacti have geographical distributions that will be negatively impacted by climate change and/or other global change stressors (Pillet *et al.*, 2022). However, because species of cacti occur across a broad range of climates, from extreme deserts to the wet tropics, the impacts of climate change and other global environmental change factors are likely to be wide ranging across species and habitats.

In this paper, we review global change threats to species of cacti that have distributions in arid to semi-arid subtropical regions. Our review focuses primarily on four global change forces: (1) increases in atmospheric CO_2_ concentrations; (2) increases in mean annual temperatures and heat waves; (3) increases in the duration, frequency and intensity of droughts; and (4) increases in competition and wildfire frequency resulting from non-native species invasion. We identify phenotypic traits within and among phylogenetic lineages that might buffer species from the effects of global environmental change. Our review will focus specifically on four primary trait syndromes: (1) stem functional morphology (including stem storage volume to photosynthetic surface area ratios); (2) rooting depths; (3) stem thermal tolerance mechanisms; and (4) plasticity in the ratio of CAM/C_3_ photosynthetic expression. We present four interrelated hypotheses that underscore the high morphological and physiological diversity among cacti taxa. First, if all else is equal, progressive increases in atmospheric CO_2_ will favour plants that in well-watered conditions, express CAM in all four phases over plants that express only phases I and III of the CAM cycle. Second, in most cases, a warmer and dryer climate will not favour plants with a high stem volume to surface area ratio (*V*:*S*), unless a high *V*:*S* is coupled with other mechanisms related to plant water balance to cope with resource limitations. Third, rooting depths vary among species of cacti, and those that are more deeply rooted are likely to be favoured over more shallow-rooted species in a hotter and drier climate, particularly where small precipitation events become less frequent relative to large events. And fourth, plants that display a suite of stem heat avoidance/tolerance strategies, including midday stem water loss from transpiration, will be favoured during extreme heat stress events.

We provide a broad range of potential priorities and solutions addressing the extinction risk of species and populations. Although emerging extinction risks from climate change will be difficult to mitigate, we present a roadmap for determining those species at risk from climate extremes, enhancing habitat quality after disturbance, approaches and opportunities for *ex situ* conservation and restoration, and the potential use of forensic tools for identifying plants that have been removed from the wild illegally and sold on open markets.

## INCREASES IN ATMOSPHERIC CO_2_ CONCENTRATIONS

There is growing evidence that globally, CAM plants are expanding in abundance and geographical distribution across dryland ecosystems (Borland *et al.*, 2009; Yu *et al.*, 2017). Yet, there is still considerable debate whether enhanced abundance and distribution are driven largely by increases in atmospheric CO_2_ or other factors, such as nitrogen deposition or warmer temperatures (especially warmer nocturnal temperatures) (Poorter and Navas, 2003; Yu *et al.*, 2017). Moreover, our understanding of increased abundance and distribution of CAM taxa comes largely from research conducted on facultative CAM species that switch between C_3_ and CAM depending on environmental stimuli. In a world experiencing rapid increases in CO_2_ and aridity, one would assume that facultative CAM brings plants the best of both worlds: C_3_ photosynthesis to take advantage of CO_2_ enrichment, and the expression of CAM to increase intrinsic water use efficiency (i.e. photosynthetic carbon uptake divided by stomatal conductance) dramatically relative to C_3_ and C_4_ plants during periods of drought and/or heat stress. However, the competitive advantage provided by CO_2_ enrichment to constitutive CAM plants, including almost all plants in the Cactaceae family, relative to other plant functional types remains largely unknown.

In order to disentangle potential benefits of CO_2_ enrichment on Cactaceae taxa and constitutive CAM plants more broadly, we must first compare the nuanced differences between four-phase and two-phase CAM expression. A common feature of many CAM plants is the expression of CAM photosynthesis at night and C_3_ photosynthesis during the early morning after sunrise or the late afternoon before sunset. The expression of CAM in all four phases (Osmond, 1978), whereby CO_2_ is acquired at night and stored as an organic acid (phase I), decarboxylated and refixed by Rubisco during the day (phase III) and accompanied by C_3_ photosynthesis in the early morning and late afternoon (phases II and IV), is most common when water is readily available and temperature stress is minimal (Borland *et al.*, 2011). Plants that express all four phases of CAM have the capacity to yield substantially higher rates of net photosynthesis over diel cycles compared with plants that express only phases I and III, but with the trade-off of operating with a reduced water use efficiency (Borland and Griffiths, 1996; Lüttge, 2006).

An outcome of the seminal work of Park Nobel and colleagues is the identification of a number of species of cacti that conduct CAM in all four phases when well watered, including *Hylocereus undatus*, *Opuntia ficus-indica* and *Stenocereus queretaroensis* (reviewed by Drennan and Nobel, 2000). When exposed to CO_2_ enrichment from 340–370 to 680–750 μmol mol^−1^, daily CO_2_ uptake increased between 34 and 152 %, and the percentage of daytime CO_2_ uptake (phases II and IV) increased between 10 and 21 % across the species of cacti listed above (Cui *et al.*, 1993; Raveh *et al.*, 1995; Nobel, 1996; Drennan and Nobel, 2000). Likewise, diurnal CO_2_ uptake increased in *Cylindropuntia imbricata*, another cactus species known to conduct CAM photosynthesis in all four phases, under enriched CO_2_ (Yu *et al.*, 2017). Importantly, *Cylindropuntia imbricata* outperformed a co-occurring C_4_ grass, *Bouteloua eriopoda*, when exposed to both drought and CO_2_ enrichment, indicating that predicted climate change conditions will favour stem succulents over C_4_ grasses in warm desert environments (Yu *et al.*, 2017).

In addition to potentially increasing daily carbon assimilation rates, elevated CO_2_ could also reduce mitochondrial respiration rates in CAM plants, which, in turn, could yield higher net photosynthetic rates and productivity. For example, long-term exposure to increased CO_2_ reduced dark respiration in *O. ficus-indica* cladodes at rates that were similar in magnitude to reductions in C_3_ plants exposed to elevated CO_2_ (Gomez-Casanovas *et al.*, 2007). However, rapidly increasing daytime and nighttime temperatures that are occurring throughout subtropical regions of the Americas could offset the lowering effects of elevated CO_2_ on mitochondrial respiration rates.

**Fig. 1.**
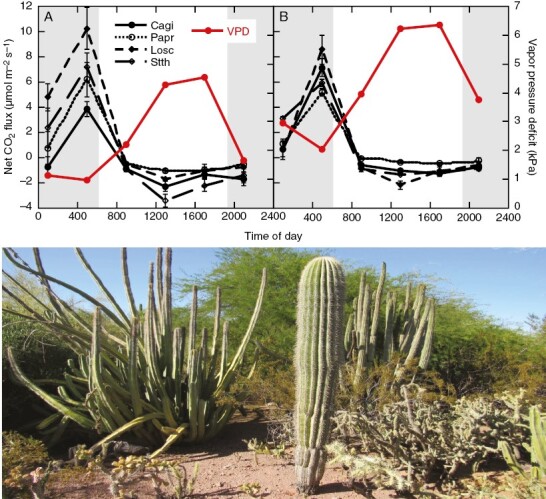
Whole-plant net diurnal CO_2_ flux and atmospheric vapour pressure deficit measured in early April of 2017 (A) and late August/early September (B) measured in well-watered, potted cactus plants native to the Sonoran Desert region, including *Carnegiea gigantea* (Cagi), *Pachycereus pringlei* (Papr), *Lophocereus schottii* (Losc) and *Stenocereus thurberi* (Stth) cacti (*n* = 5 plants per species; data are from Huber *et al.*, 2018). Error bars represent the s.e.m. Shaded regions represent nocturnal periods. The photograph shows a mature *Stenocereus thurberi* plant (back left), a young *Carnegiea gigantea* plant (front centre) and a mature *Lophocereus schottii* plant (back right) co-occurring in a common garden setting in Phoenix, AZ, USA.

Many giant cacti species that occur in some of the most arid regions of North America appear to express only phases I and III of the CAM cycle (Bronson *et al.*, 2011; Huber *et al.*, 2018), including some of the most iconic and well-distributed species of the Sonoran Desert region (Fig. 1). The limited expression of phases II and IV of the CAM cycle, even in well-watered conditions (Huber *et al.*, 2018; Fig. 1A, B) makes these species among the most water-use-efficient terrestrial plants on earth. However, the extent to which CO_2_ enrichment increases intrinsic water use efficiency, fitness or relative competitiveness with co-occurring desert C_3_ and C_4_ taxa is an open question. There is evidence that CO_2_ enrichment increases rates of nocturnal carbon uptake, although carbon uptake during phase I can quickly saturate with increasing CO_2_ concentrations (Poorter and Navas, 2003; Borland *et al.*, 2009; Sage and Stata, 2021). The positive response of phase I to CO_2_ enrichment might be related to limitations in mesophyll conductance at relatively low atmospheric CO_2_ conditions. For example, exceptionally low internal conductance values have been reported for three giant cacti species: giant saguaro (*Carnegiea gigantea*), organ pipe (*Stenocereus thurberi*) and cardón grande (*Echinopsis atacamensis*) (Williams *et al.*, 2012). Enriched CO_2_ conditions could reduce mesophyll limitations during phase I in ways that not only enhance water use efficiency, but also could enhance productivity and fitness during drought. However, the impacts of CO_2_ enrichment on desert CAM plants remain largely underexplored relative to other non-CAM taxa.

## STEM AND ROOT FUNCTIONAL MORPHOLOGY IN RELATIONSHIP TO INCREASING DROUGHT

Among the many unique features of cacti and other stem succulents is their remarkable diversity in the shape and size of their photosynthetic stems. Across the entire cactus family, stem volume relative to surface area (*V*:*S*) varies by well over two orders of magnitude (Mauseth, 2000). The broad diversity in *V*:*S* might have considerable importance for adaptation across the diverse environments where these species occur, given that stem volume determines the storage capacity of water, carbon and nutrients and that stem surface area is directly related to whole-plant photosynthetic capacity (Smith and Madhaven, 1982; Mauseth, 2000; Williams *et al.*, 2014). Stems with a low *V*:*S* should yield a relatively high rate of net carbon uptake per unit stem mass, resulting in potentially higher relative stem growth rate. However, plants with a low *V*:*S* have a lower capacity to store water, non-structural carbohydrates and other resources that would allow the maintenance of growth, photosynthesis and reproduction during periods of stress, such as drought. Therefore, variation in *V*:*S* ratio should define intrinsic biophysical and ecological trade-offs associated with stem morphology, including growth, resource use efficiency and habitat preference (Williams *et al.*, 2014; Hultine *et al.*, 2016*b*, 2019).

**Fig. 2.**
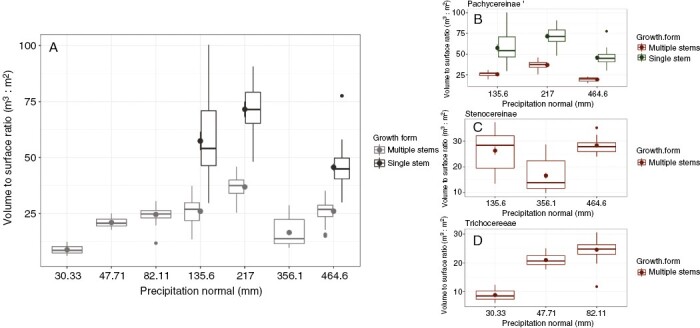
Stem volume to surface area ratio (*V*:*S*, in metres cubed per metre squared) measured in 16 species of cacti in relationship to mean annual precipitation. (A) Relationship between *V*:*S* and precipitation in all measured cacti separated into multiple-stemmed and single-stemmed species. (B, C, D) Relationship between *V*:*S* and precipitation in separate taxonomic groups, namely Pachycereinae (native to North America; B), Stenocereinae (native to North America; C) and Trichocereeae (native to South America; D). Data on stem morphology for all individual plants are available in Supplementary data Table S1. Data are previously unpublished. A description of the methods used to calculate stem *V*:*S* ratios can be found in the paper by Hultine *et al.* (2016*a*). Climate data were acquired from WorldClim, which provides raster layers at 30 arc-second (~1 km) resolution interpolated from weather station data (Hijmans *et al.*, 2005).

Given the inevitable trade-offs between photosynthetic capacity and resource storage in stem succulents, the *V*:*S* ratio should logically increase in species exposed to resource limitations and/or resource uncertainties. In a common garden in Phoenix, AZ, USA, *V*:*S* ratios of 28 columnar cacti species were generally highest in species with distributions having the most arid median climate niche (Hultine *et al.*, 2016*b*), yielding evidence that aridity is a selection agent for high resource storage capacity (Williams *et al.*, 2014). However, a more robust analysis of 16 species occurring at locations ranging in mean annual precipitation from 30 to 500 mm year^−1^ yielded no apparent relationship between stem *V*:*S* and aridity (previously unpublished; Fig. 2A). Likewise, when species were separated into phylogenetic groups, again no relationship between *V*:*S* and mean annual precipitation was detected (previously unpublished; Fig. 2B, C), except for the subtribe Trichocereeae, where mean annual precipitation was positively correlated with *V*:*S* (Fig. 2D; Supplementary data Table S1). The apparent decoupling of stem storage capacity from aridity indicates that the expression of other physiological traits enable stem succulents to cope with water limitations over extended periods.

One possible explanation is that intrinsic water use efficiency and photosynthetic expression of CAM plants are well coupled to aridity. For example, as discussed in the previous section, species of giant cacti occurring in the hottest and driest locations of the Sonoran Desert apparently do not express four-phase CAM, even when well watered (Huber *et al.*, 2018). Likewise, it is likely that arid-adapted succulent taxa can persist while CAM idling for weeks or even months in the face of drought (Nobel and Hartsock, 1983; Ting, 1985; Lüttge, 2004). Thus, it is plausible that individuals with low water storage capacity can ‘buy time’ during drought by operating in a CAM cycling mode as a way of overcoming limited water availability.

Alternatively, sensitivity to soil water deficits might be more closely related to root distribution patterns and rooting depths than to stem morphology. Unfortunately, there is a remarkable paucity of data and rooting habits of cacti plants, but evidence shows that there are considerable contrasts among species. The roots of most species of cacti are typically found no deeper than 15–30 cm below the soil surface (Cannon, 1911; Dubrovsky and North, 2002), with median rooting depths of species in the Opuntiodeae subfamily as shallow as 2.5–5.0 cm (Daugherty *et al.*, 1996). Similar to shallow-rooted herbaceous taxa, these shallow-rooted *Opuntia* species can extract moisture from rainfall events as small as 2.5 mm (Dougherty *et al.*, 1996). The high sensitivity of shallow cacti roots to small precipitation pulses is a function, in part, of determinate growth of main roots that enables rapid growth of root hairs following small precipitation pulses (Shiskova *et al.*, 2013; Rodriguez-Alonso *et al.*, 2018). However, these shallow root systems can be particularly sensitive to increases in soil temperatures as a consequence of climate warming (Schlaepfer *et al.*, 2017; Petrie *et al.*, 2020), yielding higher root respiration rates, higher root turnover rates and lower shallow soil moisture storage following precipitation events. As a consequence, populations of cacti on the warm and dry edge of a species distribution might disappear, leading to the contraction of the geographical range of many species of cacti.

Unlike the extremely shallow-rooted species in the Opuntiodeae subfamily, there is greater variation in functional rooting depths among species in the Cactoideae subfamily. For example, the mean rooting depth of the giant barrel cactus, *Ferocactus acanthodes*, occurring in the Colorado Desert of southeastern California, was reported to be 8.0 cm (Nobel, 1977). The reported rooting depth of the short-statured *Echinocereus engelmmanii*, also occurring in southeastern California, was 11.0 cm below the soil surface (Nobel *et al.*, 1991). Giant species of cacti are perhaps the deepest rooted of all cacti species, but in most cases still maintain shallow root systems relative to deep-rooted desert shrubs with dimorphic root systems. For example, *Neobuxbaumia tetetzo*, native to southern Mexico, is one of the world’s tallest species of cacti. Yet, the mean rooting depth of a 5.4-m-tall individual had extended only 25 cm below the soil surface (Valiente-Banuet *et al.*, 1991). A previous study conducted in western Sonora, Mexico showed that the maximum rooting depth of a 4.6-m-tall *Pachycereus pringlei* cactus was 1.15 m below the surface (Niklas *et al.*, 2002). Importantly, biomechanical calculations indicate that as *Pachycereus pringlei* plants grow, the shallow root systems progressively provide reduced resistance to anchoring stability and wind throw, potentially establishing an upper limit to above-ground storage capacity of water.

**Fig. 3.**
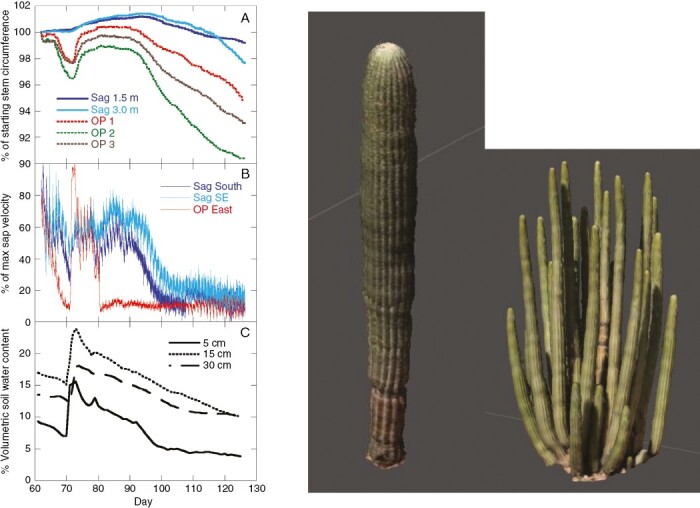
Stem circumference, root sap flux velocity and soil moisture measured every 10 min from 1 March to 5 May 2020 in the northern Sonoran Desert, in Phoenix, AZ, USA. (A) Stem circumference relative to the maximum circumference measured with DBS60 automated band dendrometers (ICT International, Armidale, NSW, Australia) on a single 4.3-m-tall *Carnegiea gigantea* plant (D, left plant) at two stem heights, and three 3-m-tall *Stenocereus thurberi* stems of the same plant (D, right plant). (B) Sap velocity measured with SFM1 heat ratio sap meter probes (ICT International) on two main lateral roots on the same *C. gigantea* plant and a main lateral root of the same co-occurring *S. thurberi* plant, relative to maximum measured velocity (for sap flux installation and data analysis methods, see Hultine *et al.*, 2003). (C) Volumetric soil water content measured at a single location near the *C. gigantea* plant using EC5 soil moisture sensors (Onset Inc., Bourne, MA, USA) installed at depths of 5, 10 and 30 cm. (D) Photogrammetric model of both species, with *C. gigantea* having an approximate surface area of 5.9 m^2^ and volume of 0.503 m^3^ and *S. thurberi* an approximate surface area of 20.8 m^2^ and volume of 0.795 m^3^.

Contrasts in rooting depths could result in considerable variation among species in sensitivity to shifting rainfall patterns, heat waves and cascading patterns of dryland ecosystem productivity and function. For example, two of the most important and iconic species of giant cacti, *Carnegiea gigantea* and *S. thurberi* (described in the previous section and in Fig. 1), co-occur naturally in some of the driest and least productive subtropical regions of the Americas. Despite their largely overlapping geographical distributions, these two species have very different stem water storage capacities and apparent functional rooting depths. Large single-stemmed species, such as *Carnegiea gigantea*, have stem volumes per unit surface area that can be ≥5-fold higher than co-occurring multi-stemmed cacti species, such as *S. thurberi* (Hultine *et al.*, 2019). The larger storage capacity in *Carnegiea gigantea* stems relative to *S. thurberi* stems should yield equally larger water residence times as long as the inputs from root water uptake and outputs through stem transpiration are equal. Indeed, seasonal patterns of stem water storage reveal that *S. thurberi* plants exhaust a much larger percentage of their stored water during the dry season compared with *Carnegiea gigantea* (Hultine *et al.*, 2019), indicating that stomatal conductance in *S. thurberi* is maintained at rates similar to *Carnegiea gigantea* during the dry season. The greater stem water storage capacity per unit photosynthetic surface area in *Carnegiea gigantea* appears to be buffered further by a deeper functional root system that can acquire soil water from deeper soil layers compared with *S. thurberi*. Continuous measurements of root sap flux coupled with continuous measurements of stem volume with stem dendrometers illustrate that the supply of water from the roots to the stems differs between species following rain events. For example, following a late winter rain event, stem volume and root sap velocity in a lateral root of a large multi-stem *S. thurberi* plant increased rapidly as soil moisture was recharged, but both also decreased rapidly as soil water in shallow layers was exhausted (previously unpublished; Fig. 3A, B). Specifically, within 10 days of the rain event, root sap velocity fell to <20 % of the maximum velocity measured following the input of rain. Conversely, stem volume and root sap velocity were far less dynamic in a 4-m-tall co-occurring *Carnegiea gigantea* plant. In fact, it took >30 days for root sap velocity to fall to <20 % of the maximum observed following the rain event in both *Carnegiea gigantea* lateral roots instrumented with heat ratio probes (Fig. 3B). These patterns indicate that mature *Carnegiea gigantea* plants achieve deeper mean rooting depths than *S. thurberi* plants. With relatively deep roots, a conservative gas exchange strategy, and stems with considerably high water storage capacities, *Carnegiea gigantea* might be relatively well buffered against the effects of drought relative to other species of giant cacti, underscoring their persistence and durability in arid regions.

## IMPACTS OF HEAT WAVES AND PLANT METABOLISM AND STRUCTURE

Unlike C_3_ and C_4_ plants, most CAM plants have a limited capacity to cool their photosynthetic tissues evaporatively at midday, when thermal stress is usually highest. Thus, depending on factors such as stem orientation, reflectance and wind speed, stem tissue temperatures can rise many degrees above ambient temperatures in sunny conditions (Smith, 1978; Smith *et al.*, 1984). Elevated stem temperatures can disrupt many plant metabolic processes. On the one hand, the carbon-concentrating CAM photosynthetic pathway eliminates or greatly reduces photorespiration in warm conditions. On the other hand, mitochondrial respiration can be greatly amplified in warm conditions, even when CAM plants are well acclimatized to heat stress (Winter *et al.*, 1986). Perhaps more pressing, thermal stress can permanently damage photosynthetic metabolism. One well-observed temperature response in photosynthetic tissues is the reduction in electron transport capacity of photosystem II, reflecting the degradation of chloroplast membranes during heat stress (Maxwell and Johnson, 2000). The critical temperature at which photosystem II begins to fail typically falls between 40 and 55 °C in most plant species (Knight and Ackerly, 2002; O’Sullivan *et al.*, 2017; Slot *et al.*, 2021), although critical temperature thresholds >60 °C have been reported in some species (O’Sullivan *et al.*, 2017). Long-term damage to chloroplast membranes could result in a catastrophic loss of photosynthetic capacity and plant carbon balance, which, over long periods, could result in plant mortality.

**Fig. 4.**
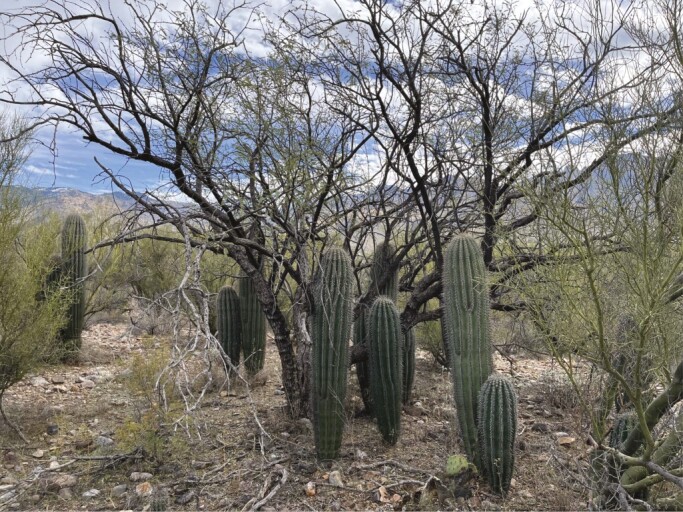
Example of nurse effects on establishment of columnar cacti in the Sonoran Desert. Here, several individuals of saguaro (*Carnegiea gigantea*) have established beneath a large velvet mesquite plant (*Prosopis velutina*) at a site in Catalina State Park, north-west of Tucson, AZ, USA. The canopy of mesquite ameliorates harsh environmental conditions, allowing for survival and recruitment of saguaro seedlings. Photograph credit: D. Williams.

With limited capacity for midday evaporative cooling, CAM succulents are potentially highly susceptible to climate change-induced heat waves, which are increasing in frequency and duration in desert and semi-desert regions of the Americas. One way in which stem succulents cope with heat waves is to establish beneath the protective canopy of nurse plants (Fig. 4). For example, midday stem temperatures of short-statured species of cacti occurring under the canopies of larger woody plants are ≤17 °C lower than those found in inter-canopy areas (Nobel *et al.*, 1986). Likewise, woody nurse plants provide critical refugia for seedlings that would otherwise be exposed to the extreme swings in temperatures that occur in desert environments (McAuliffe, 1984; Godínez-Álvarez *et al.*, 2003). However, woody plants that provide refugia for CAM succulents might themselves be under threat from global change. For example, the Sonoran Desert shrub *Ambrosia*, which serves as nurse plant for both *Carnegiea gigantea* (Turner *et al.*, 1966) and *Ferocactus acanthodes* (Franco and Nobel, 1989), experienced widespread mortality during extreme multi-year drought conditions from 1999 to 2003 (McAuliffe and Hamerlynck, 2010). Likewise, although many nurse plants, such as *Prosopis* spp., *Parkinsonia* spp. and *Olneya tesota*, avoid drought with their deep root systems, large-scale land clearing and removal has greatly reduced the aerial coverage of these species in many locations that are key for the habitat of cacti (Suzán *et al.*, 1996; Taylor, 2009). In particular, *Prosopis* spp. have been over-harvested throughout much of northern Mexico for commercial charcoal sales, reducing potential nurse plant refugia for CAM succulents throughout the region (Taylor, 2009).

**Fig. 5.**
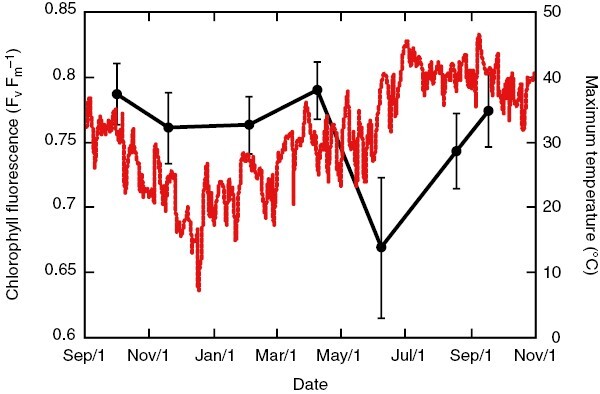
Mean *F*_v_/*F*_m_ ratios measured bi-monthly from October 2014 to 2015 on mature *Carnegiea gigantea* plants, with maximum daily air temperatures measured from a nearby weather station over the same time period in the northern Sonoran Desert in Phoenix, AZ, USA. The *F*_v_/*F*_m_ ratios were measured at all four cardinal directions at ~1.5 m above the ground surface. Measurements of *F*_v_/*F*_m_ were made at night with a FluorPen FP 100 portable fluorometer (Photon Systems Instruments, Drasov, Czech Republic). To determine *F*_v_/*F*_m_, photosynthetic tissues were exposed to an initial 30 μs (0.027 μmol m^−2^) light pulse to determine *F*_o_, followed by a 2400 μmol m^−2^ s^−1^ saturating light pulse to determine *F*_m_. Error bars represent the s.e.m.

In the absence of surrounding nurse plants, cacti must be able to tolerate exceptionally high tissue temperatures, particularly species that have massive stems with high thermal capacitance (Smith *et al.*, 1984). For example, species of barrel cactus *Ferocactus* spp. can survive with tissue temperatures reaching as high as 69 °C (Smith *et al.*, 1984). One common approach to evaluate the thermal tolerance of photosynthetic tissues is to measure variable chlorophyll fluorescence (*F*_v_/*F*_m_), defined as (*F*_m_ − *F*_o_)/*F*_m_, where *F*_m_ is the maximum fluorescence achieved by light-acclimatized photosynthetic tissues, and *F*_o_ is the fluorescence emitted by dark-acclimatized tissues (Maxwell and Johnson, 2000). In healthy photosynthetic tissues, *F*_v_/*F*_m_ is ~0.83 (unitless), with lower values indicating a potential loss of electron transport capacity of photosystem II (Maxwell and Johnson, 2000). The impact of heat stress can be illustrated by repeated measurements of *F*_v_/*F*_m_ over an annual cycle. For example, from October to April, mean *F*_v_/*F*_m_ measured on the photosynthetic stems of mature *Carnegiea gigantea* plants occurring in the very warm northern Sonoran Desert ranged from 0.76 to 0.79 (previously unpublished; Fig. 5), values that reflect a relatively robust electron transport capacity of photosystem II. However, in June, after air temperatures exceeded 45 °C, mean *F*_v_/*F*_m_ fell to 0.67 (Fig. 5), indicating that there was significant impairment of chloroplast membranes (Hüve *et al.*, 2006, 2011). Interestingly, *F*_v_/*F*_m_ did recover in August and September despite temperatures remaining >40 °C, and even exceeding 47 °C (Fig. 5), indicating that damage to chloroplast membranes was reversible and was probably supported by the rapid induction of heat shock proteins and accumulation of antioxidant enzymes and osmotic agents (Knight and Ackerly, 2003; Sharkey, 2005; Hüve *et al.*, 2006, 2011; O’Sullivan *et al.*, 2017). Whether warm-adapted plants, such as *Carnegiea gigantea*, can maintain a relatively high photosynthetic capacity when exposed to future heat wave events, which are predicted to bring much warmer conditions for longer durations, is an open question that has not yet been explored experimentally.

Heat waves and drought pose an additional threat in giant cacti, in that extreme climate stress can threaten the structural integrity of their massive stems. Immediately underneath the epidermis of cactus stems is a tissue layer termed the collenchyma that is primarily for mechanical support. These living cells contain a high concentration of pectin, a water-holding substance that when hydrated makes collenchyma tissues rigid, but flexible. The flexibility of the collenchyma allows the stem to expand when water filled and to contract over time as stored water is transpired. When giant cacti are exposed to extended drought and heat waves, the water content of collenchyma cells could fall below a critical threshold below which the mechanical support of the stem fails. Excessive heat waves and drought throughout the northern Sonoran Desert in the 2020 was probably responsible for many mature *Carnegiea gigantea* plants that toppled, resulting in a mass mortality event that is likely to be repeated regularly as desert ecosystems in the Americas become warmer and drier as predicted by global circulation models (Cayan *et al.*, 2013).

## INVASIVE SPECIES AND THE EMERGING THREAT OF WILDFIRES

Since the Holocene, the subtropical deserts of North America, including the Chihuahuan, Mojave and Sonoran Deserts, have been largely characterized by the presence of long-lived, non-fire-adapted woody shrubs and succulents interspaced within extensive areas of barren soil. The wide spacing between desert shrubs and succulents, without continuity of fine fuels in the interspaces, has historically prevented widespread destructive fires. During infrequent, wet El Niño winters, native annual forbs can occupy bare soil patches, but their rapid decomposition in early summer limits fuel loads that could trigger wildfires capable of spreading across large areas (Brooks, 1999). However, in these already parched desert regions, wildfire regimes are being amplified dramatically by the presence and exponential spread of non-native grasses. The most widespread non-native grasses include the winter C_3_ annual grasses *Bromus madritensis*, *B. rubens* and *B. tectorum* and the perennial C_4_ grasses *Cenchrus ciliaris*, *Cenchrus setaceus* and *Erogrostis lehmanniana*. These non-native, fire-adapted grasses often occupy what would otherwise be bare soil, establishing novel fuel vectors that can spread rapidly after ignition. The result of these fires is a self-perpetuating grass–fire feedback loop that progressively excludes native shrubs and succulents and results in the conversion of desert shrublands to monotypic grasslands across wide areas (D’Antonio and Vitousek, 1992; Brooks and Chambers, 2011; Betancourt, 2015; Wilder *et al.*, 2021).

**Fig. 6.**
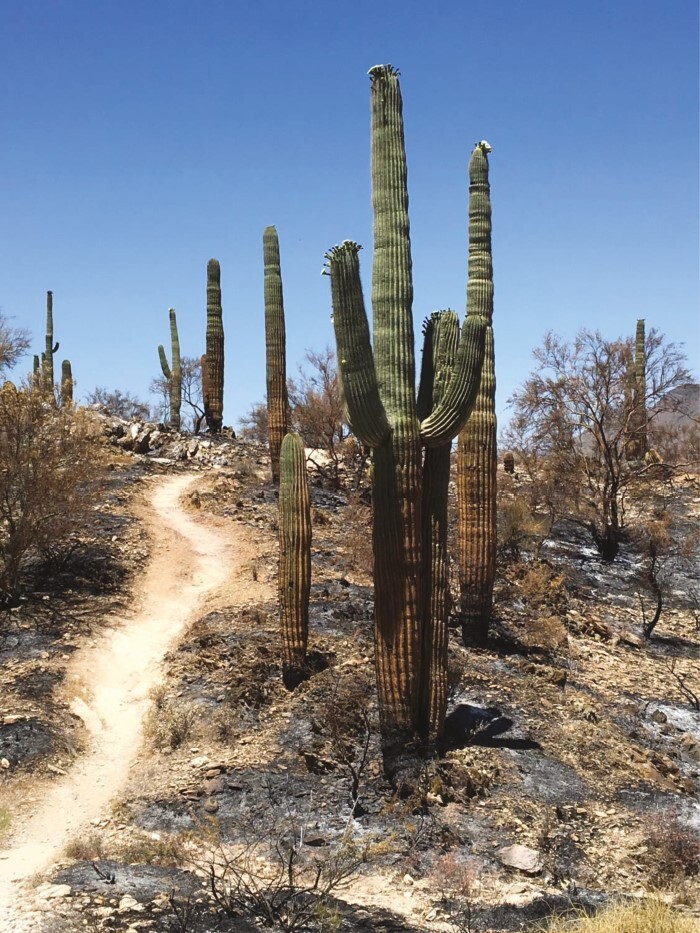
Photograph showing a severely burned population of *Carnegiea gigantea* plants following the Ocotillo Fire that burned in the northern Sonoran Desert north of Phoenix in June 2020. Photograph credit: K. Hultine.

The presence of non-native winter annual grasses in subtropical deserts has allowed wet winters to set the stage for the occurrence of destructive late spring–early summer fires that can not only lead to direct widespread losses of desert succulents, but also reduce the cover of nurse plants that form refugia for cactus seedlings (Bracamonte *et al.*, 2017). The extremely dry and hot summer that was preceded by an unusually wet winter in 2020 in the northern Sonoran Desert illustrates conditions promoting non-native grasses and their influence on desert succulents. Winter rains in 2019–2020 triggered a massive *Bromus rubens* flush northeast of Phoenix, AZ, USA, which fuelled a 783 km^2^ fire that scorched *Carnegiea gigantea*-dominated vegetation (Wilder *et al.*, 2021). Further south, expansive *Cenchrus ciliaris* cover helped to fuel a 486 km^2^ fire during the summer of 2020 that burned desert shrublands in the Santa Catalina Mountains north of Tucson, AZ, USA (Wilder *et al.*, 2021). In both these fires, and in other smaller fires that occurred in the region in 2020, thousands of *Carnegiea gigantea* plants were burned (Fig. 6). One year after the 486 km^2^ fire near Tucson, the mortality of *Carnegiea gigantea* occurring within the footprint of the fire was 18 %, with almost all dead plants having a height of ≤2 m (Wilder *et al.*, 2021). However, the full impact of the fire is currently unknown because *Carnegiea gigantea* mortality after severe burning of surface tissues can take ≤6 years (Esque *et al.*, 2004). Moreover, the impact of the fire was even more destructive to smaller-statured desert succulents, such that species of *Opuntia*, *Cylindropuntia* and *Echinocactus* rarely survived after 1 year in the burned locations (Wilder *et al.*, 2021).

The threat of fire to CAM succulents is not limited to subtropical regions of the Americas. The presence of non-native grasses in tropical dry forests, which host some of the highest cactus diversity in the world, is altering fire frequencies and intensity. For example, cacti growing in a tropical dry forest ecosystem in Puerto Rico were more likely to burn in areas dominated by invasive grasses and native shrubs than those growing under a closed tree canopy in the absence of grasses (Carrera-Martinez *et al.*, 2020). However, the emerging threat of wildfires to native cacti in tropical dry forests is not driven exclusively by grass invasion and climate change. For example, most of the fires that now occur in the dry Chaco of northern Argentina are from human ignition sources to clear shrubs and promote grass productivity in pastures (Boletta *et al.*, 2006; De Marzo *et al.*, 2022). These fires often spread into neighbouring forests that host a high diversity of species of cacti, including the iconic giant cacti *Stetsonia coryne* and *Echinopsis terscheckii*. The promotion of fire and other forest-clearing initiatives in tropical dry forests in Mexico (Mesa-Sierra *et al.*, 2022) and throughout the Gran Chaco of South America is rapidly reducing habitat in regions with some of the highest densities of threatened and endangered species of cacti in the world (Goettsch *et al.*, 2015). Additionally, the intensity of human-caused fires appears to be exacerbated by drought (Fisher *et al.*, 2012; Argañaraz *et al.*, 2015), indicating that human land use and climate change will synergistically reduce prime habitat for endangered species of cacti in both North and South America.

## CONSERVATION SOLUTIONS AND PRIORITIES

Recent assessment by the IUCN reveals that species of cacti are among the most threatened taxonomic groups in the world, regardless of life form or habitat (Goettsch *et al.*, 2015; Hultine *et al.*, 2016*b*; Pillet *et al.*, 2022). With >30 % of all species of cacti listed as either threatened or endangered (Goettsch *et al.*, 2015), many species are at extreme risk of extinction owing to a wide range of factors, including habitat loss, invasive species, climate change and the illegal horticultural trade of rare and endemic species (Goettsch *et al.*, 2015; Pillet *et al.*, 2022). Mitigating the ongoing and emerging threats to cacti will require not only strong policy initiatives and international cooperation, but also new and creative approaches to conservation. Below is a synthesis of many (but by no means all) potential approaches that could be undertaken to improve the capacity for practitioners to protect species of cacti in the wild.

### Monitoring the impacts of climate change through enhanced networking

Given that cacti occur across a remarkably diverse range of habitats, it is nearly impossible to make a general prediction of how climate change will impact cacti as a whole. Nevertheless, there is considerable evidence that climate change will increase the extinction risk of many species of cacti throughout the New World (Pillet *et al.*, 2022). In the absence of the exceptional global cooperation needed to combat climate change, there are no obvious solutions for mitigating its impacts on species of cacti at risk. However, the vast global network of existing academic institutions and botanical gardens could be used to evaluate species and populations at risk. Botanical gardens provide living collections that are usually distributed such that they mimic common garden settings (Miller-Rushing *et al.* 2007; Donaldson, 2009). Some of these living collections contain a remarkable variety of plant species from different geographical areas, providing rare opportunities to monitor a wide range of physiological and phenotypic traits of plants growing in common conditions. For example, of the 3243 botanical gardens profiled worldwide by Botanic Gardens Conservation International (bgci.org), 114 include members of Cactaceae as a major part of their living collections. Many of these gardens are located within one of the primary centres of cactus diversity in Mexico and the southwestern USA (Hultine *et al.*, 2016*b*). A research programme focused on documenting, improving and better curating the living collections of these botanical gardens could be used to establish a reciprocal common garden network that spans a broad mean, annual maximum and minimum temperature gradient. Future programmes should aim to expand the reciprocal common garden concept by assuring that whenever possible, common genotypes are identified and propagated at multiple gardens to study relationships between phenotypic trait expression and climate (Hultine *et al.*, 2016*b*).

### Community-wide management of non-native, fire-adapted grasses

As restoration ecologists and practitioners know, the widespread removal of destructive invasive species can be a daunting task that is rarely successful without broad support across agencies, communities and institutions. As mentioned earlier, non-native, fire-adapted grasses have dramatically altered the fire cycle of many dryland areas, resulting in massive mortality mosaics of succulent plants and less suitable habitat for regermination (Shyrock *et al.*, 2014; Bracamonte *et al.*, 2017; Carrera-Martinez *et al.*, 2020; Wilder *et al.*, 2021). In southern and central Arizona, USA, initiatives to map and remove the non-native C_4_ fire-adapted grass *Cenchrus ciliaris* across large parcels of private and public lands have been spearheaded by stakeholders working with institutions such as the Sonoran Desert Museum near Tucson (https://www.desertmuseum.org/buffelgrass/) and the Central Arizona Conservation Alliance, which supports regional parks in the Phoenix area (https://cazca.org). Efforts by these institutions are providing a road map to improve management of native lands and conservation of succulent taxa at risk.

In the tropical dry forests in Mexico, Central America and the Dry Chaco of South America, fire prevention is not dependent on controlling non-native, fire-adapted grasses but on policies and social programmes to prevent forest clearing by fire and/or mechanical approaches for pastures and agricultural production. Tropical dry forests continue to disappear rapidly around the globe, and in the Americas these forests host a remarkable diversity of cacti that are threatened and endangered. However, some fire prevention programmes have yielded successful outcomes. For example, in Costa Rica, fire prevention strategies in protected areas have fostered a quantifiable increase in forest structure and species richness (Powers *et al.*, 2009). Likewise, the Argentine Forest Law, passed in 2007, has had some success in preventing fires and reducing land clearance in the Argentine Chaco region (De Marzo *et al.*, 2022). Likewise, in Mexico, the federally supported and publicly available biodiversity informatics database (CONABIO; Soberón, 2022) is being used widely to track wildfires in real time (Ressl *et al.*, 2009), thereby increasing the capacity to respond rapidly to wildfires in some of the most biologically sensitive regions of the country. Without these and other fire-prevention campaigns, the threat of fire to the biodiversity of cacti will most probably increase exponentially with climate change and land use to support human enterprise.

### Protecting and promoting nurse plant refugia

Warmer and drier climatic conditions coupled with episodic land disturbance are increasing the dependence of cacti on nurse plant refugia. As a consequence, programmes to restore cactus habitat in most areas require sustaining and/or restoring woody plant cover over large areas. Many large-scale efforts are in place, such as in Mexico, where the Federal Government has set a target of restoring 8 million ha of degraded forest land, with a dual goal of forest conservation and alleviation of poverty (Torres-Rojo *et al.*, 2019). These efforts include targeting tropical dry forests that host numerous species of cacti (Mesa-Sierra *et al.*, 2022). Additionally, nurse plant performance and nurse plant facilitation of cacti can be amplified through symbiotic associations between plants and mycorrhizae, because most perennial desert plant species appear to form symbiotic associations with mycorrhizal fungi, in particular arbuscular mycorrhizae (Bloss, 1985; Carrillo-Garcia *et al.*, 2002; Gardezi *et al.*, 2022). The restoration of subtropical regions to promote woody nurse plant restoration, particularly following disturbance, could be enhanced if seeding of native plants is restored with inoculation with endemic soil microbes, including arbuscular mycorrhizae (Korb *et al.*, 2004; Shackelford *et al.*, 2021). For example, intense fires not only kill both succulent and associated woody nurse plants but can also destroy associated arbuscular mycorrhizae. However, reinoculation of burned areas using nearby unburned soils can increase the success of native vegetation (Korb *et al.*, 2004). Thus, reinoculation of highly disturbed soils might be a viable restoration tool to set the stage for rapid reconstruction of cactus habitat after disturbance.

### Managing the illegal horticultural trade of rare and endemic species of cacti

To this point, this review has not covered the impact of the illegal horticultural trade of cacti, which is, arguably, the most pressing factor threatening cacti throughout the Americas (Goettsch *et al.*, 2015). Stemming the illegal horticultural trade of plants and seed will require not only enormous multinational cooperation, but also creative, ‘next-generation’ approaches to track individual plants removed from the wild. For example, the application of barcoding developed from genetic markers specific to a given species might be possible given the accessibility of next-generation sequencing techniques together with the availability of already published full species genomes (Copetti *et al.*, 2017). A barcoding database also could aid substantially in the identification of wild-collected specimens that might not present sufficient morphological characters for proper identification when recovered from illegal markets or ports of entry (Yessen *et al.*, 2011; Hultine *et al.*, 2016*b*).

Additionally, stable isotopes have been used in forensic applications to isolate the provenances (origins) of various plant materials (Ehleringer *et al.*, 2008; Bartelink *et al.*, 2016). For example, the stable isotopic composition of precipitation has been used to identify the provenance of plant products from spatially explicit maps of the natural abundance of stable isotopes (i.e. isoscapes) over ecological and geological systems to generate predictive models (West *et al.*, 2007; Bowen, 2010). There is the potential to use the isoscape framework to target illegal trade of rare plants, including species of cacti (Snyder *et al.*, 2022). Given that many cactus species are endemic to small areas, their tissues should record only a small range of values of δ^18^O and δ^2^H in the wild and should be predictable using isoscape modelling approaches (West *et al.*, 2007). Thus, stable isotopes of cactus tissues, such as spines, could be analysed from plants obtained from points of entry along international borders to detect whether they were collected illegally from wild populations or cultivated legally in greenhouse facilities (Snyder *et al.*, 2022). Although this isoscapes approach is untested, it could provide one new avenue for securing the future of cacti taxa in the wild.

## CONCLUSIONS

Species of cacti are among the most recognizable and ecologically important of all terrestrial plant groups on the planet. Yet, somewhat paradoxically, they are also among the most threatened of all extant taxa, largely owing to intensive land clearance, increased fire frequency and the removal of plants from endemic wild populations to trade illegally across the globe (Goettsch *et al.*, 2015). Climate change could synergistically enhance ongoing threats to the biodiversity of cacti or, in some cases, counter other threats depending on whether populations respond negatively or favourably to elevated CO_2_, increased temperatures (particularly nighttime temperatures) and altered hydrological cycles relative to C_3_ or C_4_ taxa (Sage and Stata, 2021). In areas where cacti are negatively impacted by human activities, ecosystems will be likely to experience a significant alteration in the flow of energy and water across multiple scales. In turn, these alterations could have profound cascading impacts on a wide range of ecohydrological processes, animal biodiversity and local economies that depend on services from water-limited ecosystems.

## SUPPLEMENTARY DATA

Supplementary data are available online at https://academic.oup.com/aob and consist of the following. Table S1: cactus species, growth form, subtribe, geographical location, stem diameter, stem cross-sectional area, stem perimeter, stem volume to surface area ratio, mean annual precipitation and mean annual temperature of source location extracted from WoldClim.

mcad040_suppl_Supplementary_Materials
